# C22:0- and C24:0-dihydroceramides Confer Mixed Cytotoxicity in T-Cell Acute Lymphoblastic Leukemia Cell Lines

**DOI:** 10.1371/journal.pone.0074768

**Published:** 2013-09-09

**Authors:** Michael W. Holliday Jr., Stephen B. Cox, Min H. Kang, Barry J. Maurer

**Affiliations:** 1 School of Medicine Cancer Center, Texas Tech University Health Sciences Center, Lubbock, Texas, United States of America; 2 Research and Testing Laboratory, Lubbock, Texas, United States of America; 3 Departments of Cell Biology & Biochemistry and Pharmacology, Texas Tech University Health Sciences Center, Lubbock, Texas, United States of America; 4 Departments of Cell Biology & Biochemistry, Pediatrics and Medicine, Texas Tech University Health Sciences Center, Lubbock, Texas, United States of America; University of Louisville, United States of America

## Abstract

We previously reported that fenretinide (4-HPR) was cytotoxic to acute lymphoblastic leukemia (ALL) cell lines *in vitro* in association with increased levels of *de novo* synthesized dihydroceramides, the immediate precursors of ceramides. However, the cytotoxic potentials of native dihydroceramides have not been defined. Therefore, we determined the cytotoxic effects of increasing dihydroceramide levels via *de novo* synthesis in T-cell ALL cell lines and whether such cytotoxicity was dependent on an absolute increase in total dihydroceramide mass versus an increase of certain specific dihydroceramides. A novel method employing supplementation of individual fatty acids, sphinganine, and the dihydroceramide desaturase-1 (DES) inhibitor, GT-11, was used to increase *de novo* dihydroceramide synthesis and absolute levels of specific dihydroceramides and ceramides. Sphingolipidomic analyses of four T-cell ALL cell lines revealed strong positive correlations between cytotoxicity and levels of C22:0-dihydroceramide (ρ = 0.74–0.81, *P* ≤ 0.04) and C24:0-dihydroceramide (ρ = 0.84–0.90, *P* ≤ 0.004), but not between total or other individual dihydroceramides, ceramides, or sphingoid bases or phosphorylated derivatives. Selective increase of C22:0- and C24:0-dihydroceramide increased level and flux of autophagy marker, LC3B-II, and increased DNA fragmentation (TUNEL assay) in the absence of an increase of reactive oxygen species; pan-caspase inhibition blocked DNA fragmentation but not cell death. C22:0-fatty acid supplemented to 4-HPR treated cells further increased C22:0-dihydroceramide levels (*P* ≤ 0.001) and cytotoxicity (*P* ≤ 0.001). These data demonstrate that increases of specific dihydroceramides are cytotoxic to T-cell ALL cells by a caspase-independent, mixed cell death mechanism associated with increased autophagy and suggest that dihydroceramides may contribute to 4-HPR-induced cytotoxicity. The targeted increase of specific acyl chain dihydroceramides may constitute a novel anticancer approach.

## Introduction

The synthetic retinoid N-(4-hydroxyphenyl)retinamide (fenretinide, 4-HPR) has demonstrated cytotoxic activity *in vitro* to cell lines of multiple cancer types, including T-cell acute lymphoblastic leukemia (ALL) [1–4]. Mechanisms of action of 4-HPR include increased reactive oxygen species (ROS) levels in certain cancer cell lines [4–9]. 4-HPR also stimulated the *de novo* sphingolipid pathway leading to a time- and dose-dependent increase of dihydroceramides in multiple model systems [9–15].

**Figure 1:**
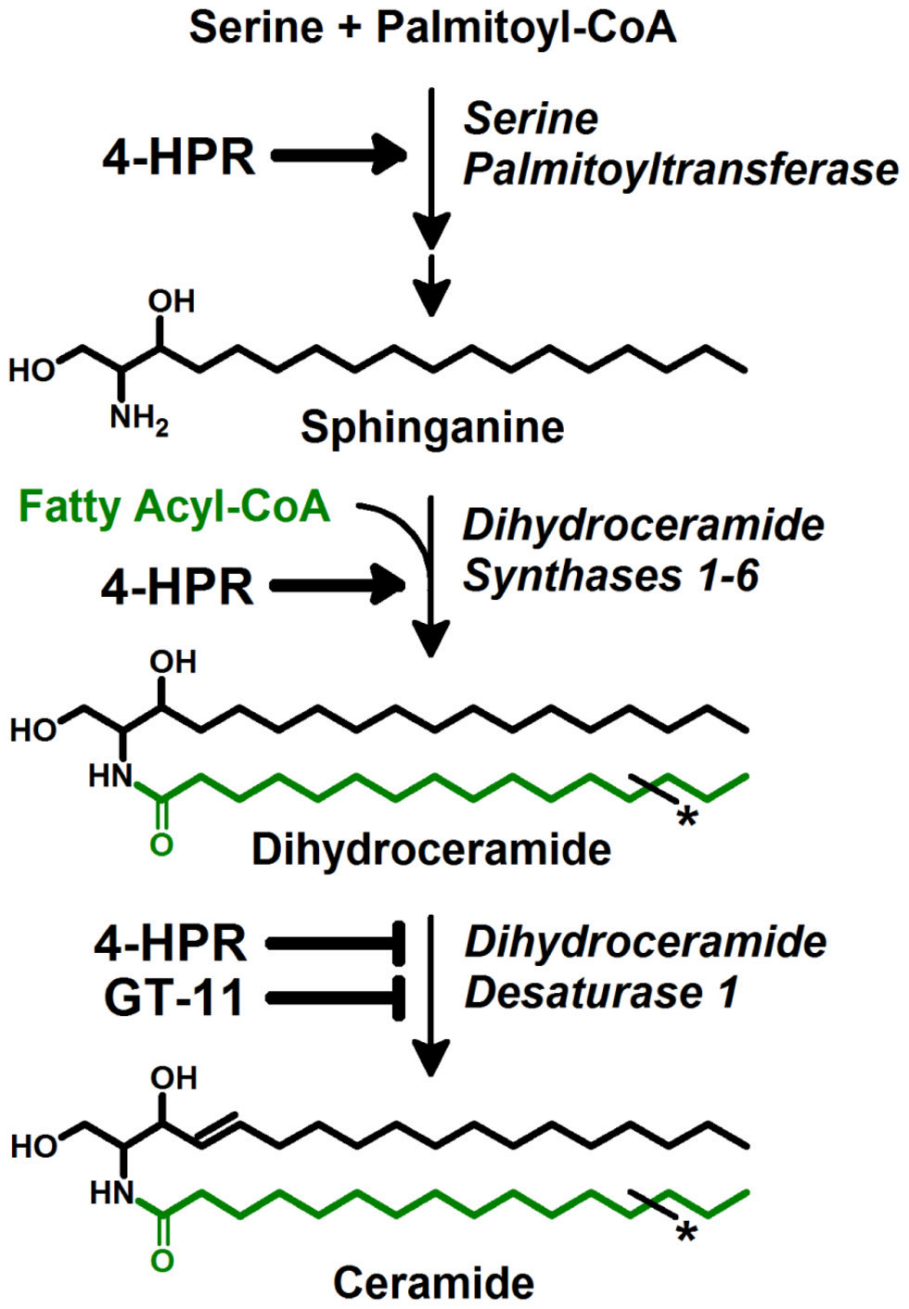
Schematic of the *de novo* ceramide pathway. Rate-limiting enzyme, serine palmitoyltransferase (SPT), condenses serine and palmitoyl-CoA to 3-ketosphinganine, which is subsequently reduced to sphinganine. Dihydroceramide synthases 1-6 (CerS 1-6), each utilizing a preferred subset of fatty acid-derived acyl-CoAs, add a fatty acyl chain (green) to sphinganine to produce dihydroceramides. Dihydroceramide desaturase (DES1) converts dihydroceramides to ceramides by introduction of a 4,5-trans double bond into the sphinganine backbone of dihydroceramide. 4-HPR stimulates both SPT and CerS in certain cancer cell lines. Both 4-HPR and GT-11, a synthetic ceramide derivative, inhibit DES1. Asterisks (*) indicate variable carbon length and saturation.

Dihydroceramides are the direct precursors of ceramides in the mammalian *de novo* sphingolipid pathway (Figure 1). The rate-limiting enzyme of the pathway, serine palmitoyltransferase (SPT), regulates sphinganine synthesis. The family of dihydroceramide synthases (CerS 1-6) acylate sphinganine with a fatty acyl chain to form a dihydroceramide, with each CerS utilizing a preferred subset of fatty acyl-CoAs whose acyl chains vary both in carbon length (14- to 30-) and degree of saturation [16–18]. Carbons 4 and 5 of the sphinganine backbone of the dihydroceramide are reduced by dihydroceramide desaturase (DES1) to yield the corresponding ceramide [19]. We previously reported that 4-HPR increased the activities of serine palmitoyltransferase and dihydroceramide synthase in a neuroblastoma cell line resulting in an increased ‘ceramides’ fraction and that 4-HPR increased ceramides coincident with cytotoxicity in a dose- and time-dependent manner in acute lymphoblastic leukemia cell lines [2,20]. Recent work with more advanced methodologies has demonstrated that 4-HPR specifically increases dihydroceramides due to concurrent inhibition of dihydroceramide desaturase 1 (DES1) [13–15].

Extensive literature supports that intracellular ceramides have death-signaling properties, but such studies have rarely distinguished the relative activity of individual ceramide species [21,22]. In contrast, there is much less data on the bioactive properties of dihydroceramides, the saturated precursors of ceramides. Such investigations have relied mainly on the use of exogenous, synthetic, cell penetrant, very short saturated acyl chain (C2:0 – C8:0) dihydroceramides [23–27], although several more recent reports have reported the possible involvement of native acyl chain dihydroceramides in cell death processes [28–33]. Given the observed association between increased dihydroceramides and 4-HPR-induced cytotoxicity, we hypothesized that the cytotoxic activities of artificial very short-acyl chain dihydroceramides are not representative of native acyl chain dihydroceramides, and that the cytotoxic potential of dihydroceramides is acyl chain length and/or saturation dependent.

The difficulty in directly assessing the cytotoxic potentials of native acyl chain dihydroceramides over-induced by pharmacological agents (i.e. ‘ceramide-stress’) arises from the technical challenge of exogenously delivering such large amphipathic sphingolipids into cells. Further, the approach of increasing native dihydroceramides through overexpression of the various ceramide synthases is limited by the intracellular availability of precursor substrate, sphinganine, and the use of multiple fatty acyl-CoA’s by any given ceramide synthase family member (Figure 1). Therefore, an aim of the current study was to develop a biochemical system to mimic pharmacologically-induced ceramide stress (i.e., selectively increase the levels of native acyl chain dihydroceramides and ceramides via *de novo* synthesis). To achieve this, cells were exogenously supplemented with a minimally-cytotoxic concentration of sphinganine to increase the *de novo* synthesis of dihydroceramides, and with GT-11, a competitive inhibitor of DES1, to decrease the conversion of the resulting dihydroceramides to their corresponding ceramides, thus broadly mimicking the dihydroceramide-increasing effects of 4-HPR [34]. α-Cyclodextrin was then employed as a water soluble carrier to deliver selected fatty acids to sphinganine ± GT-11 treated cells to increase the synthesis of the corresponding acyl chain dihydroceramide [35]. We also sought to distinguish whether dihydroceramide cytotoxicity was associated with an increase in the *total mass* of dihydroceramides, irrespective of acyl chain composition, or with an increase in levels of *specific* acyl chain dihydroceramides. Cell death was characterized by measures of autophagy, apoptosis, caspase dependency and ROS levels. We also determined how the levels of dihydroceramides in 4-HPR-treated cells were affected by supplementation with specific fatty acids and the effects of such supplementation on 4-HPR-induced cytotoxicity.

Our results indicate that increased levels of certain, but not all, native acyl chain dihydroceramides are cytotoxic to T-cell ALL cell lines. Our results also suggest that supplementation of dihydroceramide-increasing anticancer agents with specific fatty acids, whether administered separately or by incorporation into a formulation vehicle, might result in increased efficacy, possibly in a cancer or cancer type-specific manner.

## Materials and Methods

### Materials

Sphinganine ([2S,3R]-2-aminooctadecane-1,3-diol) and GT-11 (N-[(1R,2S)-2-hydroxy-1-hydroxymethyl-2-(2-tridecyl-1-cyclopropenyl)ethyl]octanamide) were from Avanti Polar Lipids (Alabaster, AL, USA), and prepared in ethanol at 10 mM and 1 mM, respectively. ABT-737 (4-[4-[[2-(4-chlorophenyl)phenyl]methyl]piperazine-1-yl]-N-[4-[[(2R)-4-(dimethylamino)-1-phenylsulfanylbutan-2-yl]amino]-3-nitrophenyl]sulfonylbenzamide) was from Santa Cruz Biotechnology (Santa Cruz, CA, USA). Boc-D-FMK was from Imgenex (San Diego, CA, USA). Fenretinide, (4-HPR, (2E,4E,6E,8E)-N-(4-hydroxyphenyl)-3,7-dimethyl-9-(2,6,6-trimethylcyclohexen-1-yl)nona-2,4,6,8-tetraenamide), graciously provided by the National Cancer Institute (NCI) Developmental Therapeutics Program (DTP) of the National Institutes of Health (NIH, Bethesda, MD, USA), was prepared in ethanol (10 mM). Chloroform (ethanol-stabilized) and other solvents were obtained from Sigma Aldrich (St. Louis, MO, USA) or Fisher Scientific (Pittsburg, PA, USA). α-Cyclodextrin (Acros Organics, Geel, Belgium) was dissolved (15 mM) in RPMI-1640 medium (Invitrogen, Carlsbad, CA, USA). Sphingolipid standards were from Avanti Polar Lipids. Radiolabeled ^3^H-sphinganine, docosanoic [1-C^14^] and tetracosanoic acids [1-C^14^] (50 mCi/mmol) were from American Radiolabeled Chemicals (St. Louis, MO, USA). Fatty acids (FA) were purchased from Sigma Aldrich, and included the following: tetradecanoic acid, hexadecanoic acid, octadecanoic acid, (Z)-ocadec-9-enoic acid, icosanoic acid, (Z)-icos-11-enoic acid, docosanoic acid, (Z)-docos-13-enoic acid, tetracosanoic acid and (Z)-tetracos-15-enoic acid. Fatty acids were dissolved in a solution of methanol/chloroform (1:2, v:v) at 10 mM and stored in PFTE-capped borosilicate vials.

### Fatty acid solubilization

Fatty acids were solubilized by modification of Singh and Kishimoto [35]. Fatty acid stock was added to a glass flask and dried under nitrogen (~10 PSI). α-Cyclodextrin (15 mM in RPMI-1640) was added at 27.3 mL/µmol FA. The sealed flask was then sonicated three times for 5 minutes each using a Branson 2510 Bath Sonicator (30°C). The fatty acid solution was then sterilized by filtration (0.22 µm PVDF filter, EMD Millipore, Billerica, MA, USA) and diluted 3 parts FA to 1 part RPMI-1640 medium. The efficiency of fatty acid solubilization and fatty acid cellular uptake was demonstrated using ^14^C-C22:0- and ^14^C-C24:0-fatty acid tracers, thin layer chromatography separation, and liquid scintillation counting (data not shown); fatty acid solutions were prepared to ≥15 µM, and used at a final concentration of 5 µM in whole cell culture medium.

### Cell culture

T-cell ALL cell lines, CCRF-CEM and p53 gene mutated MOLT-4, were from American Type Culture Collection (Manassas, VA, USA) and grown at 5% O_2_/5% CO_2_ and 20% O_2_/5% CO_2_, respectively. The T-cell ALL cell lines COG-LL-317h and COG-LL-332h were obtained from the TTUHSC Cancer Center Cell Repository and grown at 5% O_2_/5% CO_2_. Cell line identities were verified by the Children’s Oncology Group Cell Culture and Xenograft Core using the AmpF/STR Identifiler system (Applied Biosystems, Carlsbad, CA, USA), and mycoplasma testing was performed. Cell lines were maintained in RPMI-1640 medium supplemented with 10% fetal bovine serum (FBS, Invitrogen, USA) in humidified 37°C incubators. For all experiments, cells were seeded at 2.5x10^5^ cells/mL in RPMI-1640 supplemented with 15% FBS, with a final FBS content of 10% after addition of fatty acids and/or drugs in serum free medium. Control treatments consisted of fatty acid and drug vehicles.

### Cytotoxicity assay

Cytotoxicity was measured using DIMSCAN (Bioimaging Solutions, Inc., San Diego, CA, USA), a semi-automated, fluorescence-based, digital imaging microscopy method with a 4-log dynamic range of detection [36]. Briefly, digital image thresholding of cells treated with a fluorophore (fluorescein diacetate) and quencher (2′4′5′6′-tetrabromofluorescein) enabled capture and quantification of live cell fluorescence via a CCD camera. For cytotoxicity assays, cells were seeded (2.5x10^4^ cells/well) into a 96-well flat bottom plate (BD-Falcon) in 100 µL of whole medium one hour prior to addition of the various treatment reagents added in 50 µL and assayed at +48 hours of treatment exposure. Cytotoxicity of each treatment condition was measured in two or more separate experiments except where otherwise indicated.

### LC/MS/MS analysis of sphingolipids

For sphingolipid analysis, cells were seeded (7.5x10^6^ cells) one hour before treatment. After six hours of treatment, cells were washed with ice-cold phosphate-buffered saline (PBS) and stored at -80°C for sphingolipid analysis. Each treatment was prepared in triplicate and repeated a minimum of two times. Sphingolipids were separated using an Agilent 1200 HPLC and determined by ESI/MS/MS performed on a Applied Biosystems SCIEX 4000 QTRAP Hybrid Triple Quadrupole/Linear Ion Trap mass spectrometer, operating in a multiple-reaction monitoring, positive ionization mode as described previously with modifications [37]. Specifically, 50 µL of a solution (1 pM) of internal sphingolipid standards (including C17-sphingosine, C17-sphinganine, C17-sphingosine-1-phosphate, and C17-ceramide) was added to each cell pellet. Cells were extracted twice with ethyl acetate/isopropyl alcohol/water (60:28:12; v:v). Sample was divided for quantitative and lipid phosphate analyses. For LC/MS/MS, sample was re-dissolved in mobile phase A (ammonium formate [1 mM] and formic acid [0.2%] in methanol). Samples were injected (10 µL) and separated on a Spectra C8SR, 150 x 3.0 mm, 3 µm particle size column (Peeke Scientific, Redwood City, CA) using gradient-elution with mobile phase A and B (ammonium formate [2 mM] and formic acid [0.2%] in water). Data acquisition, peak integration and analyte quantitation were performed using ABI/SCIEX Analyst 1.4.2 Software. Sphingolipid data were normalized to lipid phosphate as previously described [38]. Briefly, lipids were extracted using the method of Bligh and Dyer [39]. Disposable borosilicate tubes (Kimble Chase, Vineland, NJ, USA) were used so that acid washing was not necessary. Sample organic phase was isolated and a known volume was separated to a new tube and dried at 80°C. Phosphate standards and dried samples were then heated with ashing buffer (water:10 N H_2_SO_4_:70% HClO_4_ [40:9:1]) at 160°C overnight. Samples were subsequently incubated with ammonium molybdate and ascorbic acid, and absorbance (820 nM) was measured using a SpectraMax M2e (Molecular Devices, Silicon Valley, CA, USA).

### Immunoblotting

Treated cells were washed with PBS and lysed on ice with RIPA Buffer (Thermo, Fisher) supplemented with protease inhibitors (EMD Millipore) and 2% Triton-X100 (to ensure complete LC3B-II solubilization). Cellular membranes were disrupted by sonication. Protein content was measured using the BCA assay as directed (Thermo, Fisher). Proteins were separated using Bis-Tris (Invitrogen) gels. Proteins were transferred to PVDF membranes (Invitrogen) using a Semi-Dry Western Blot method (Thermo, Fisher). Blots were blocked in 5% milk/TBST and the lower molecular weight regions were probed with the either anti-LC3B (Cell Signaling [CS], Beverly, MA) or anti-caspase 3 (CS) antibodies. Higher molecular weight regions of each blot were probed with anti-β-actin (Santa Cruz Biotechnology [SBT]), which served as loading control. The secondary antibodies used were HRP-anti-rabbit (CS) and HRP-anti-mouse (SBT). LC3B-transfected, HEK-293 cell lysate was included as a positive control (Novus Biologicals, Littleton, CO). Blots were developed using ECL Substrate (Invitrogen) and visualized using a VersaDoc MP 5000 (Bio-Rad, Hercules, CA, USA) equipped with a 50 mm, f1.4 fixed focal length lens. Data were analyzed using Quantity One Software (Bio-Rad).

### Assay of DNA fragmentation by flow cytometry

Treated cells were stained using the Apo-Direct kit (a TUNEL (Terminal deoxynucleotide transferase dUTP Nick End Labeling) assay variant)(BD Pharmingen, San Diego, CA, USA) as directed. Cells (including compensation controls) were analyzed on an LSR II Custom Flow Cytometer System (BD Biosciences, San Jose, CA, USA). Doublet discrimination and data analysis was performed using FlowJo (v10, Tree Star, Inc., Ashland, OR, USA) software for Macintosh.

### Detection of reactive oxygen species (ROS) and mitochondrial depolarization by flow cytometry

For ROS assay, treated cells were stained as previously described with 2’, 7’-dichlorofluorescein diacetate (125 µM; Sigma Aldrich) [4]. 4-HPR (10 µM) and H_2_O_2_ (120 mM) were employed as positive controls. Each treatment was assayed in triplicate in two independent experiments. Mitochondrial depolarization was assayed using the MitoProbe JC-1 kit (Invitrogen), and analyzed on an LSR II Custom Flow Cytometer System in single experiments. Data were analyzed using FacsDiva (v6.0) software.

### Real-time PCR

Total RNA was extracted with the RNeasy Mini-Kit (Qiagen, Valencia, CA, USA), with QIAshredder columns employed for improved cell lysis efficiency. RNA quality and quantity was measured by spectrophotometry using a NanoDrop 1000 (Thermo, Fisher). First-strand cDNA synthesis was performed using the High Capacity cDNA Reverse Transcription Kit (Applied Biosystems). TaqMan Gene Expression Master Mix and Primer/Probe sets (Applied Biosystems) were used as recommended with 100 ng of cDNA per reaction (20 µL), and real-time quantitative PCR was performed on an Applied Biosystems 7900HT Fast Real-Time PCR System employing the standard method. The following TaqMan primer/probe sets (Applied Biosystems) were used: GAPDH, Hs03929097_g1; HPRT1, Hs02800695_m1; CerS1, Hs00242151_m1; CerS2, Hs00371958_g1; CerS3, Hs00698859_m1; CerS4, Hs00226114_m1; CerS5, Hs00908757_m1; CerS6, Hs00826756_m1. Real-time PCR samples were assayed in triplicate and data were analyzed using the 2^(-ΔΔ^_CT_^)^ method [40]. Data were normalized to GAPDH and calibrated to the CerS1 mRNA of CCRF-CEM cells.

### Statistical Analysis

T-tests (two-tailed) were used to compare sphingolipid levels and cytotoxicity (quantified as the surviving fraction) of treatment groups with those of control groups. Analysis of variance was used to determine whether addition of fatty acids significantly affected the cytotoxicity of sphinganine ± GT-11. Spearman’s non-parametric rank correlation was used to quantify relationships between cytotoxicity and sphingolipid analyte levels. SigmaPlot 11 and Microsoft Excel 2011 were employed for statistical testing. Correlation analyses were performed in the R Statistical Environment (2.15.0).

## Results

### Dihydroceramides were cytotoxic to T-cell ALL cell lines

**Figure 2:**
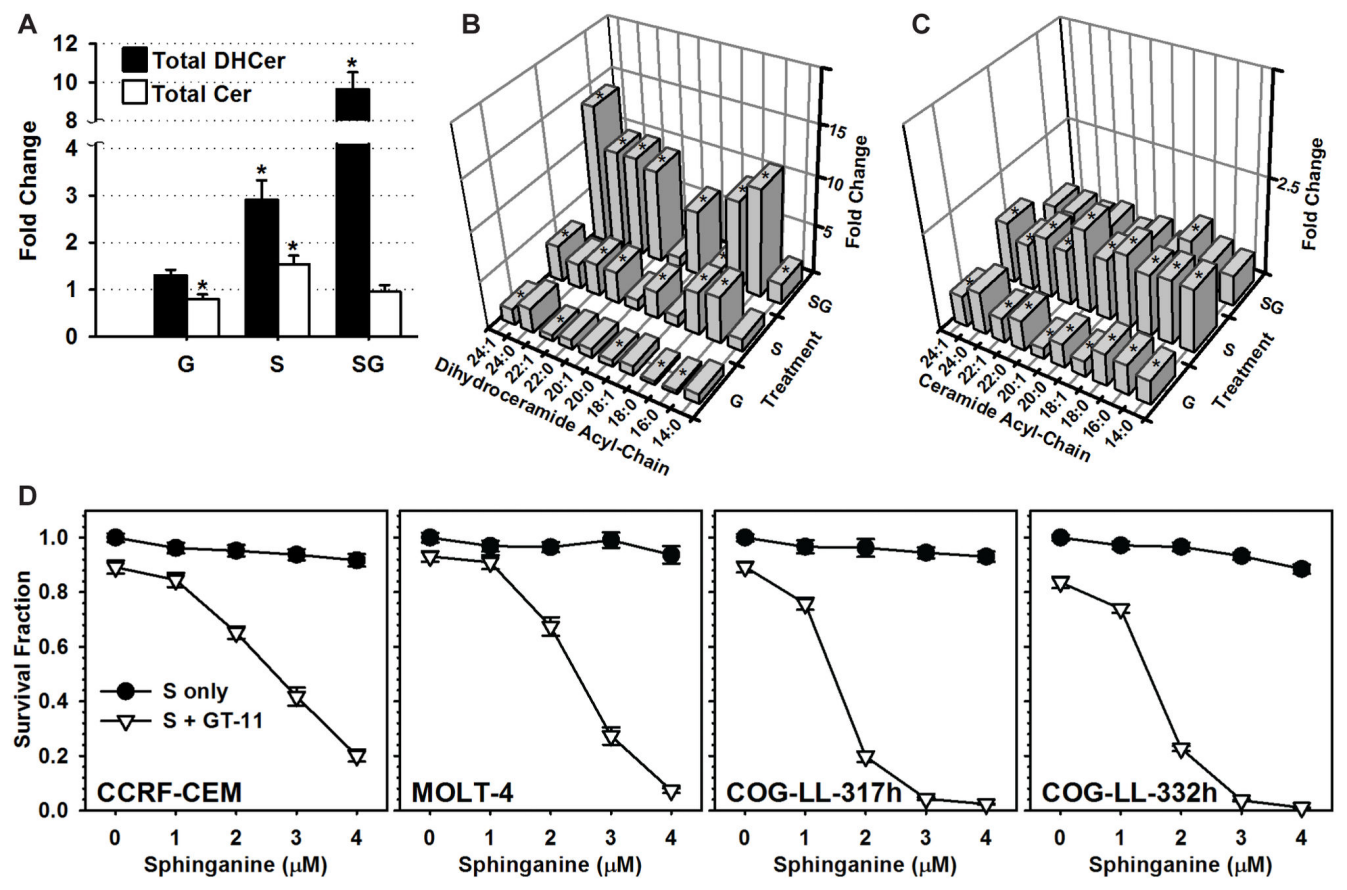
Effects of sphinganine and GT-11 on dihydroceramides, ceramides and cytotoxicity. *A*-*C*) CCRF-CEM cells were treated with sphinganine (4 µM, S) ± GT-11 (0.5 µM, G) for six hours and sphingolipids analyzed. *A*) Total dihydroceramides (DHCer) and ceramides (Cer) were normalized to drug/fatty acid vehicle-treated cells (control) and plotted as fold change (Y-axis). Error bar, propagated standard deviation. Individual dihydroceramides (*B*) and ceramides (*C*) were normalized to control and plotted as fold change (Z-axis). Dihydroceramides and ceramides are identified by acyl chain (x:y), where x is the number of carbons and y is the number of double bonds in the acyl chain (X-axis). Significant (*A*, *P* ≤ 0.001*; B* and C, P ≤ 0.05) fold changes between treatment and vehicle control levels are indicated by asterisks (*). *D*) Cytotoxicity of sphinganine and GT-11. Indicated cell lines were treated with sphinganine (0-4 µM) and/or GT-11 (0.5 µM). The cytotoxicity of GT-11-alone is represented by the sphinganine (0 µM) data point. Cytotoxicity assayed by DIMSCAN cytotoxicity assay at +48 hours. Data were normalized to vehicle-treated control and plotted as Survival Fraction (Y-axis). Error bar, SEM. Sphinganine + GT-11 resulted in significantly increased (*P* ≤ 0.001) cytotoxicity relative to sphinganine-only for all concentrations and across all cell lines.

Incorporation of exogenous sphinganine into *de novo* synthesized intracellular dihydroceramides/ceramides was demonstrated utilizing ^3^H-sphinganine (not shown). Treatment of CCRF-CEM cells with sphinganine resulted in a 2.9-fold increase (*P* ≤ 0.001) in total dihydroceramides (Figure 2A), a 1.5-fold increase (*P* ≤ 0.001) of total ceramides and significant increases (*P* ≤ 0.05) in each of the individual ceramides assayed (Figure 2C). Addition of DES1-inhibitor, GT-11, to sphinganine (i.e., sphinganine + GT-11) resulted in a 9.6-fold increase (*P* ≤ 0.001) of total dihydroceramides, including significant increases (*P* ≤ 0.05) in all individual dihydroceramides except C20:1-dihydroceramide (Figure 2B). Sphinganine + GT-11 also resulted in a decrease of both total (*P* ≤ 0.001) and individual ceramides (*P* ≤ 0.02) relative to sphinganine-alone.

Sphinganine-alone (1–4 µM) and GT-11-alone (0.5 µM) were minimally cytotoxic (Figure 2D). However, the combination of sphinganine + GT-11 increased cytotoxicity (*P* ≤ 0.001) in a sphinganine concentration-dependent manner in each of the cell lines tested. Thus, the combination of sphinganine and GT-11 increased dihydroceramide levels in CCRF-CEM cells, and increased cytotoxicity in all four T-cell ALL cell lines.

### Addition of specific fatty acids increased levels of dihydroceramides and ceramides

**Figure 3:**
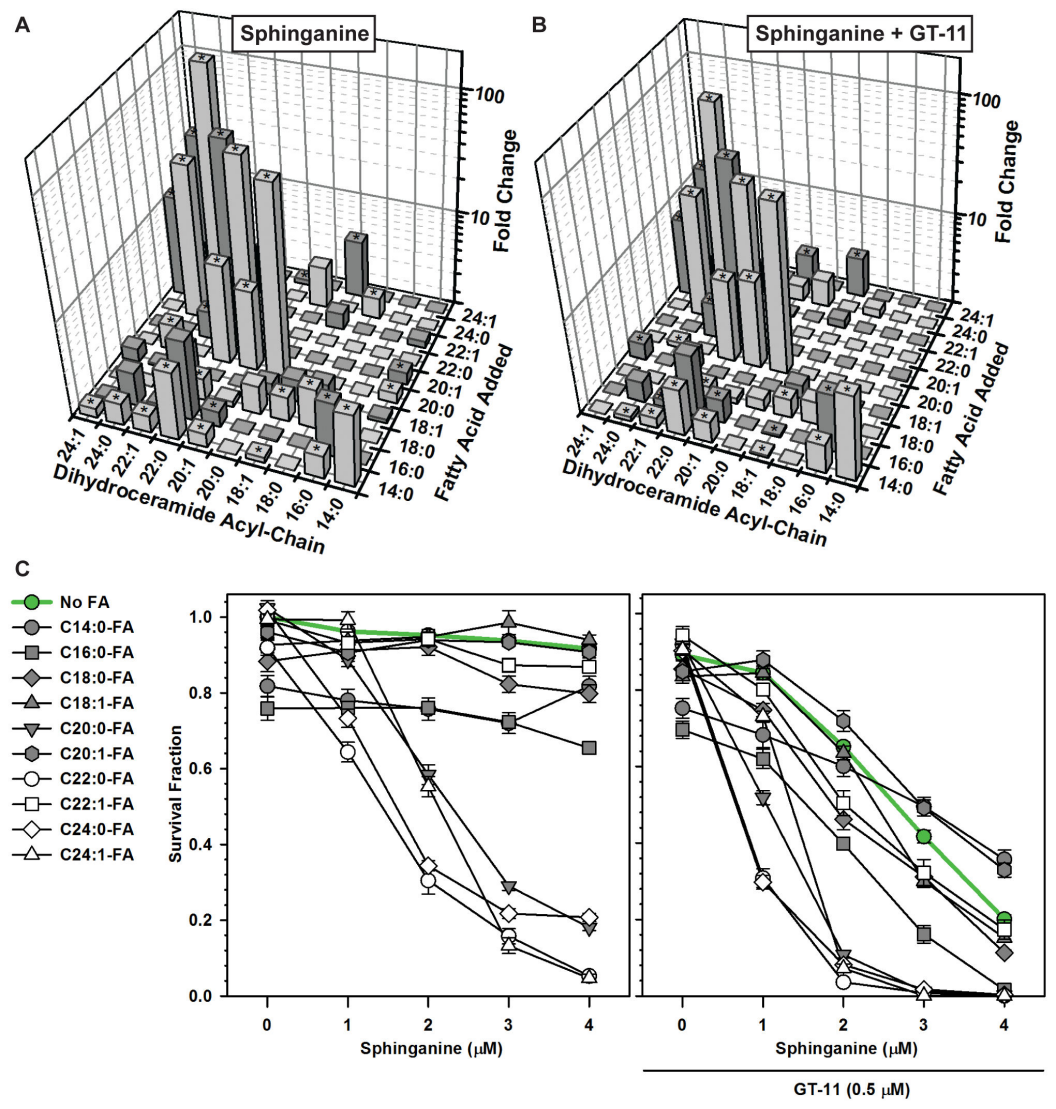
Effects of specific fatty acids on sphinganine ± GT-11-induced dihydroceramides and cytotoxicity. *A*, *B*) Effects on dihydroceramides. CCRF-CEM cells were treated with (*A*) sphinganine (1 µM), or (*B*) sphinganine (1 µM) + GT-11 (0.5 µM), and supplemented with the indicated fatty acids (5 µM) for six hours, followed by sphingolipid assay. To evaluate the effects resulting from addition of each fatty acid, data for (*A*) and (*B*) were normalized either to cells that received sphinganine-only with no fatty acid supplementation (*A*), or to sphinganine + GT-11 without fatty acid (*B*), and plotted as fold change (Z-axis). Fatty acids are identified by x:y, where x is the number of carbons and y is the number of double bonds in the fatty acid chain (Y-axis). Significant (*P* ≤ 0.05) differences from sphinganine-only are indicated by asterisks (*). *C*) Effects on cytotoxicity. CCRF-CEM cells were treated with sphinganine-only (0-4 µM), or sphinganine (0-4 µM) + GT-11 (0.5 µM), supplemented with the indicated fatty acids (5 µM). Cytotoxicity assayed by DIMSCAN cytotoxicity assay at +48 hours. Data were normalized to control and plotted as survival fraction (Y-axis). Error bar, SEM. Data, grouped by sphinganine dose, were analyzed by one-way ANOVA.

To increase levels of specific acyl chain dihydroceramides and ceramides, CCRF-CEM cells were treated with sphinganine ± GT-11 and supplemented with individual fatty acids (FA) (C14:0-, C16:0-, C18:0-, C18:1-, C20:0-, C20:1-, C22:0-, C22:1-, C24:0- or C24:1-FA). Fatty acids were solubilized with α-cyclodextrin. Incorporation of solubilized fatty acid into *de novo* ceramides was demonstrated using ^14^C-tetracosanoic acid (C24:0-FA) (not shown). Of the fatty acids tested, C14:0-, C16:0, C18:0, C20:0-, C22:0-, C22:1-, C24:0- and C24:1-FA supplemented to sphinganine significantly increased levels (*P* ≤ 0.05) of the corresponding acyl chain dihydroceramides relative to sphinganine with no fatty acid (Figure 3A). C14:0-, C16:0-, C20:0-, C22:0-, C22:1-, C24:0- and C24:1-FA supplemented to sphinganine + GT-11 significantly increased levels (*P* ≤ 0.05) of the corresponding acyl chain dihydroceramides relative to sphinganine + GT-11 with no fatty acid (Figure 3B). C14:0-, C16:0-, C18:0-, C20:0-, C20:1-, C22:0-, C22:1-, C24:0- and C24:1-FA supplemented to sphinganine significantly increased levels (*P* ≤ 0.05) of the corresponding acyl chain ceramides relative to sphinganine-alone in CCRF-CEM cells (Figure S1). Certain fatty acids increased the corresponding ceramide when given in combination with sphinganine + GT-11, likely due to incomplete DES1 inhibition in the presence of high dihydroceramide substrate levels, but not to the extent observed when the fatty acid was combined with sphinganine-alone. Certain fatty acids also resulted in concurrent increases of longer and/or desaturated acyl chain dihydroceramides and ceramides due to intracellular modification of the fatty acids by elongases and desaturases prior to acylation of sphinganine [41]. As an example, when C22:0-FA was added to sphinganine + GT-11, in addition to the observed 29-fold increase (*P* ≤ 0.001) of C22:0-dihydroceramide, there was a 3-fold (*P* ≤ 0.01) and 19-fold (*P* ≤ 0.001) increase of C22:1- and C24:0-dihydroceramides, respectively, compared to sphinganine + GT-11 without fatty acid.

### Addition of specific fatty acids increased cytotoxicity

Certain, but not all, fatty acids increased (*P* ≤ 0.001) the cytotoxicity of sphinganine ± GT-11 (Figure 3C) in a sphinganine concentration-dependent manner. In general, longer chain fatty acids increased cytotoxicity to a greater degree than did shorter chain fatty acids.

### Levels of C22:0- and C24:0- dihydroceramides positively correlated with cytotoxicity

Since the intracellular metabolism of certain fatty acids resulted in an increase of multiple dihydroceramides and/or ceramides, to identify individual level-dependent cytotoxic relationships, quantitative sphingolipid levels and cytotoxicity data were analyzed using the Spearman’s non-parametric rank correlation. Correlations were evaluated using sphingolipids assayed at +6 hours of treatment and cytotoxicity assayed at +48 hrs, as serial assay demonstrated that minimal further changes in sphingolipid profiles occurred at later time points (not shown). Additionally, sphingolipid data used for correlation analyses were taken at a fixed exogenous sphinganine concentration (1 µM) corresponding to a modest cell kill fraction to minimize possible confounding secondary changes in sphingolipid profiles resulting from death in large fractions of the cells. Of all sphingolipid analytes, only levels of C22:0- (ρ = 0.74, *P* ≤ 0.001) and C24:0- (ρ = 0.84, *P* ≤ 0.001) dihydroceramides demonstrated strong positive correlations with cytotoxicity (for scatter plots, see Figure S2). No other consistent positive or negative correlations were observed between cytotoxicity and other sphingolipid analytes (total or individual dihydroceramides, total or individual ceramides, sphingoid bases, including sphinganine, or phosphorylated sphingoid bases). The absolute amounts of the dihydroceramides which correlated with cytotoxicity, and the amounts of their corresponding ceramides, are shown in Table 1. C22:1-dihydroceramide levels increased to a larger absolute amount than C22:0-dihydroceramide, but did not correlate with cytotoxicity; therefore, this species served as a negative control to exclude effects based on increase of total dihydroceramide mass irrespective of acyl chain.

**Table 1:** Absolute sphingolipid levels.

		**Treatment**				
**Species**	**Basal**	**S**	**S + FA**	**S + G**	**S + G + FA**	**Fatty Acid**
C22:0-DHCer	0.13 ± 0.03	0.93 ± 0.09	45 ± 4	2.0 ± 0.2	57 ± 4	C22:0-FA
C22:0-Cer	0.37 ± 0.04	0.6 ± 0.1	7.7 ± 0.4	0.37 ± 0.04	4.6 ± 0.3	C22:0-FA
C22:1-DHCer	0.18 ± 0.01	1.2 ± 0.1	53 ± 5	2.3 ± 0.2	73 ± 7	C22:1-FA
C22:1-Cer	0.05 ± 0.01	0.16 ± 0.03	2.5 ± 0.4	0.07 ± 0.01	1.05 ± 0.07	C22:1-FA
C24:0-DHCer	0.16 ± 0.05	0.61 ± 0.04	78 ± 6	1.4 ± 2	99 ± 10	C24:0-FA
C24:0-Cer	0.37 ± 0.05	0.40 ± 0.06	4.4 ± 0.8	0.28 ± 0.04	2.6 ± 0.4	C24:0-FA

Effects of specific fatty acids on absolute levels of corresponding dihydroceramides and ceramides in CCRF-CEM cells treated with sphinganine ± GT-11. Absolute levels (pmol sphingolipid/nmol lipid phosphate) are shown (± SEM) of intracellular sphingolipids from untreated cells (Basal), or cells treated with sphinganine (S, 1 µM) or sphinganine and GT-11 (G, 0.5 µM) with and without supplemented with fatty acid (FA). The specific fatty acids used to supplement cells are indicated in the fifth column.

### Characterization of dihydroceramide-induced cell death mechanisms

**Figure 4:**
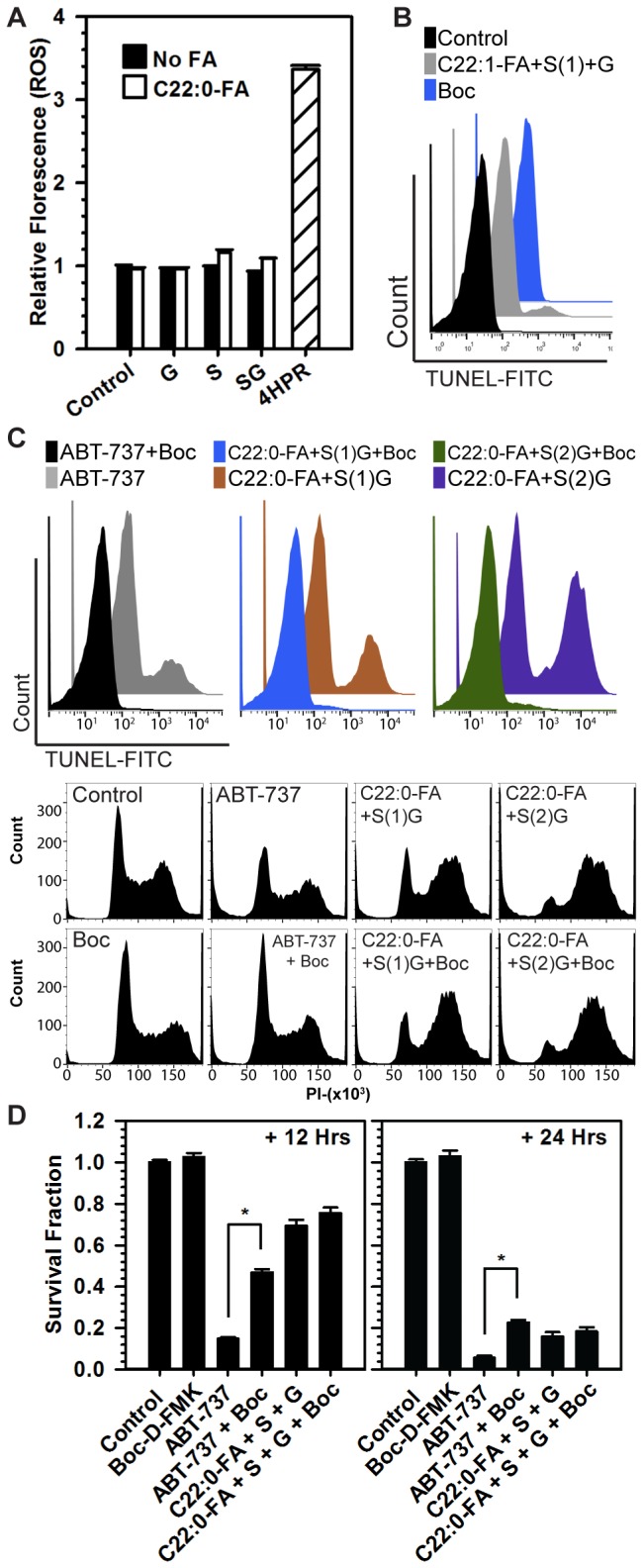
Mechanisms of C22:0-dihydroceramide induced cell death. *A*) Reactive oxygen species levels in sphinganine and/or GT-11 treated CCRF-CEM cells supplemented with or without C22:0-fatty acid. CCRF-CEM cells were treated with sphinganine (1 µM) and/or GT-11 (0.5 µM), both with and without C22:0-FA (5 µM). 4-HPR (10 µM) and H_2_O_2_ (120 mM, not shown) were employed as positive controls. Cells were stained with 2’, 7’-dichlorofluorescein diacetate and fluorescence analyzed after 6 hours by flow cytometry. Data were normalized to control. Error bars, SEM. *B* & *C*) Effect of pan-caspase inhibition on TUNEL positivity. CCRF-CEM cells were pre-treated with pan-caspase inhibitor, Boc-D-FMK (80 µM), or DMSO (final concentration = 0.33%, Boc-D-FMK vehicle control), for one hour prior to treatment with ABT-737 (1 µM, positive control), or C22:0-FA plus sphinganine (1 or 2 µM, S) + GT-11 (0.5 µM, G). C22:1-FA plus sphinganine (1 µM) + GT-11 (0.5 µM, G) and Boc-D-FMK alone included as controls. Cells analyzed by TUNEL assay at +24 hrs. Shown are histograms representative of three separate experiments. Histograms are of indicated treatments analyzed by PI counter-stain of TUNEL samples. *D*) Effect of pan-caspase inhibition on cytotoxicity. CCRF-CEM cells were pre-treated with pan-caspase inhibitor, Boc-D-FMK (80 µM, Boc), for one hour prior to treatment with ABT-737 (1 µM), C22:0-FA plus sphinganine (2 µM, S) + GT-11 (0.5 µM, G). Cytotoxicity assessed at +12 and +24 hrs by DIMSCAN cytotoxicity assay and represented as Survival Fraction. Asterisks (*) represent significant (*P* ≤ 0.05) effects of Boc treatment.

**Figure 5:**
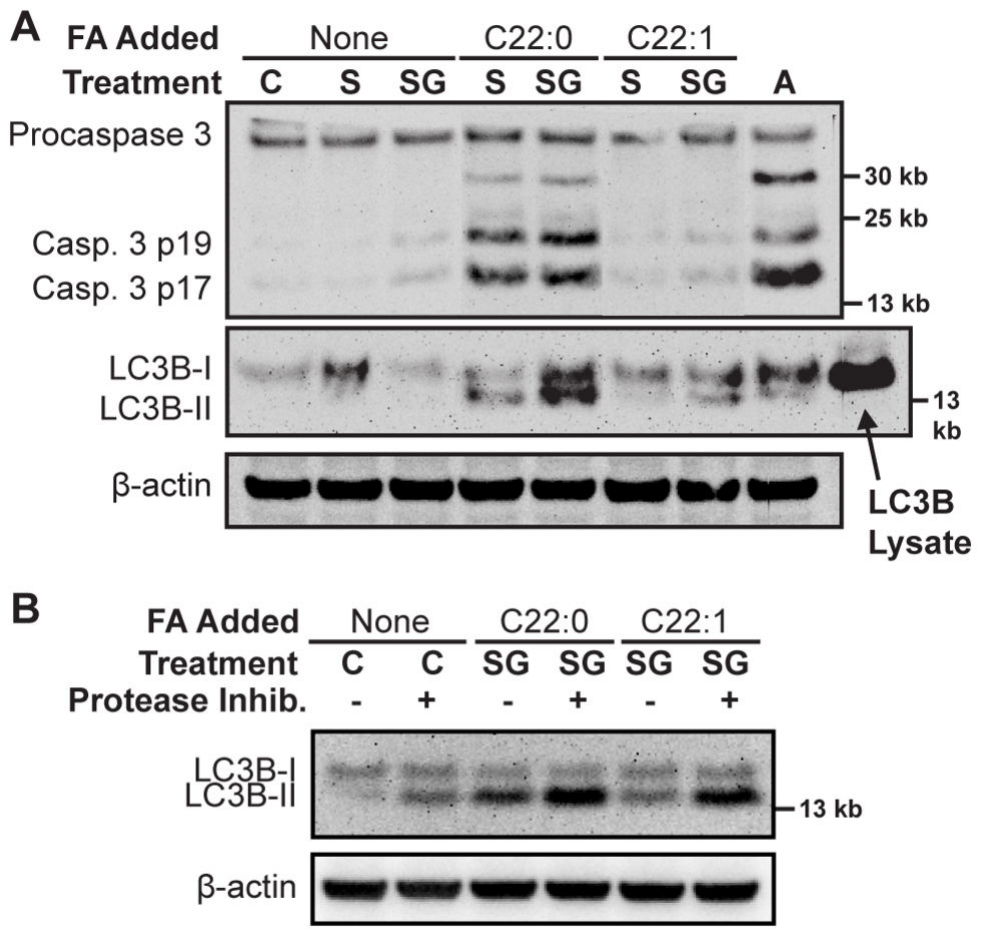
Caspase cleavage and LC3B-I/II turnover. *A*) CCRF-CEM cells treated with drug/fatty acid vehicle (C), sphinganine (1 µM, S), or sphinganine (1 µM) + GT-11 (0.5 µM) (SG), were supplemented with the indicated fatty acids (5 µM). After 12 hours, total proteins were extracted and procaspase-3 (35 kb), activated caspase-3 (17/19 kb), and LCB3-I/II (14/16 kb), were detected by immunoblotting. β-Actin served as loading control. Treatment with pan-Bcl-2 inhibitor, ABT-737 (1 µM, A), and LC3B-transfected, HEK-293 cell lysate, were used as positive controls. C22:1-fatty acid was used as a negative control for C22:0-fatty acid. Lanes rearranged to ease interpretation. Data representative of three separate experiments are shown. *B*) Assessment of LC3B-II flux. CCRF-CEM cells were pretreated with or without protease inhibitors (Pepstatin-A and E64d) and treated as described. After 12 hours, total proteins were extracted and LC3B-I/II analyzed by immunoblotting. Data representative of three separate experiments are shown, except for C22:1-fatty acid.

Because 4-HPR increases reactive oxygen species (ROS) levels in association with cytotoxicity in certain cancer cell lines, ROS levels were measured in treated CCRF-CEM and MOLT-4 cells to determine if an increase of dihydroceramides was contributory to ROS increase. Compared to treatment with 4-HPR, an increase in ROS was not observed with sphinganine and/or GT-11 treatments, with or without C22:0-FA, at doses that increased dihydroceramides and conferred cytotoxicity (Figure 4A). Mitochondrial depolarization proceeded concurrently with, but did not precede, cell death in cells treated with sphinganine/GT-11/C22:0-FA (not shown). To assess markers of apoptosis, DNA fragmentation was assayed by terminal deoxynucleotidyltransferase duty nick end labeling (TUNEL) and caspase cleavage by immunoblotting. Cells treated with C22:1-FA plus sphinganine and GT-11 exhibited minimal TUNEL positivity at +24 hrs (Figure 4B and Figure S3). In contrast, treatment with C22:0-FA plus sphinganine ± GT-11 increased TUNEL positivity relative to controls (Figure 4C). Increased procaspase-3 cleavage was increased by C22:0-FA plus sphinganine ± GT-11 (combinations that increased cytotoxicity), but procaspase-3 cleavage was not increased by C22:1-FA plus sphinganine ± GT-11 (combinations that minimally effected cytotoxicity)(Figure 5A). To determine whether cytotoxicity was dependent on caspase-mediated DNA fragmentation, the effect of a pan-caspase inhibitor, Boc-D-FMK, on C22:0-dihydroceramide-mediated cell death was assessed. The addition of Boc-D-FMK to ABT-737, a known inducer of apoptosis in ALL cell lines [4], abrogated both ABT-737-induced TUNEL positivity and reduction in the G1 cell population (Figure 4C), as well as reduced ABT-737-induced cytotoxicity (Figure 4D). In contrast, while Boc-D-FMK abrogated C22:0-FA plus sphinganine + GT-11 induced TUNEL positivity (Figure 4C), it did not decrease C22:0-FA plus sphinganine + GT-11 induced cytotoxicity, suggesting that under conditions of caspase inhibition cell death proceeded via a non-apoptotic mechanism (Figure 4D). Interestingly, the proportion of cells treated with C22:0-FA plus sphinganine + GT-11 that were in G1 significantly reduced relative to controls, while the proportion of cells in G2 remained similar or increased.

Lipidation of LC3B-I to LC3B-II, a marker of increased autophagic vacuolization, increased in cells treated with C22:0-FA plus sphinganine ± GT-11 to a much greater extent than with C22:1-FA (Figure 5A). To determine whether LC3B-II level elevation was due to increased LC3B-I lipidation (flux) or to decreased LC3B-II degradation, cells were pretreated with protease inhibitors of LC3B-II degradation, E64d and pepstatin-A (each at 10 µg/mL), prior to exposure to C22:0-FA or C22:1-FA plus sphinganine + GT-11. E64d and pepstatin-A pretreatment resulted in a marked increase of LC3B-II in cells compared to controls for both C22:0-FA and C22:1-FA, suggesting an increase of autophagic flux (Figure 5B). Interestingly, addition of 3-methyladenine (0.1-10 mM), a putative autophagy inhibitor, neither increased nor decreased the cytotoxicity of C22:0-FA plus sphinganine ± GT-11 in CCRF-CEM cells (not shown), suggesting that autophagy was not directly linked to the cell death mechanism.

### C22:0- and C24:0-dihydroceramides positively correlated with cytotoxicity in other ALL cell lines

**Figure 6:**
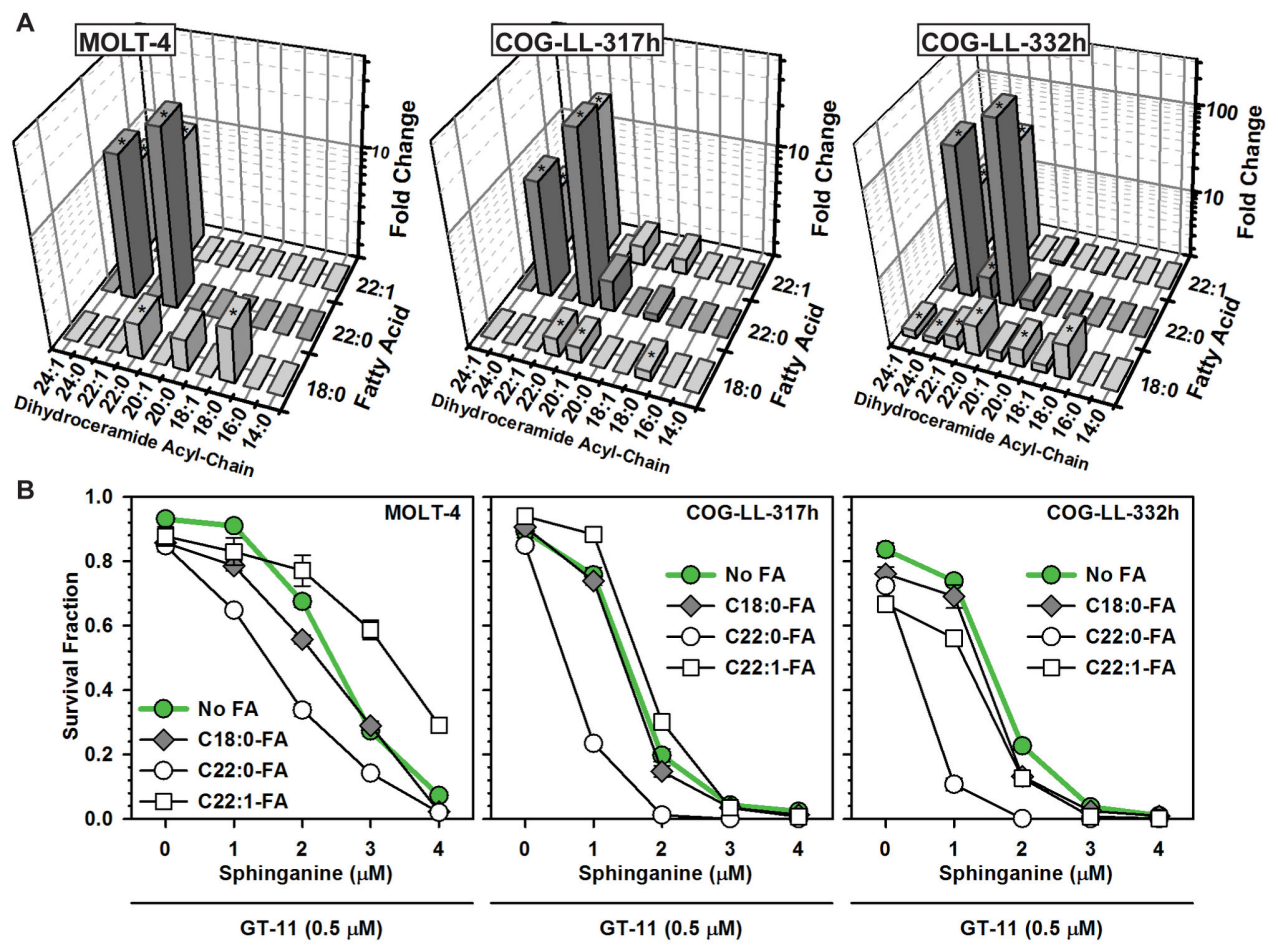
Effects of specific fatty acids on sphinganine + GT-11-induced dihydroceramide accumulation and cytotoxicity. *A*) Effects of fatty acids on dihydroceramide levels. MOLT-4, COG-LL-317h, and COG-LL-332h, cell lines were treated with sphinganine (1 µM) + GT-11 (0.5 µM) supplemented with the indicated fatty acid (5 µM) for six hours and sphingolipids analyzed. Data are normalized to cells that received sphinganine + GT-11 without fatty acid supplementation and dihydroceramide levels plotted as fold change (Z-axis). Significant (*P* ≤ 0.05) fold change differences are indicated by asterisks (*). *B*) Effects of fatty acids on cytotoxicity. Cell lines were treated with sphinganine (0-4 µM) ± GT-11 (0.5 µM) and supplemented with C18:0-, C22:0- or C22:1-fatty acids (5 µM). Cytotoxicity assessed at +48 hours by DIMSCAN cytotoxicity assay. Data were normalized to controls and plotted as Survival Fraction (Y-axis). Error bar, SEM.

Given the results in CCRF-CEM cells, the effects of C18:0-, C22:0- and C22:1-FA supplemented to sphinganine ± GT-11 were tested in three additional T-cell ALL cell lines (MOLT-4, COG-LL-317h and COG-LL-332h). C22:1-FA was also tested because, while minimally affecting the cytotoxicity of sphinganine + GT-11 in CCRF-CEM cells, it resulted in absolute levels of C22:1-dihydroceramide similar to those of C22:0-dihydroceramide after treatment with C22:0-FA, thus effectively serving as a negative control for a possible non-specific (acyl chain-independent) mass effect of increased dihydroceramides on cytotoxicity. C22:0-FA added to sphinganine + GT-11 increased C22:0-dihydroceramide in MOLT-4, COG-LL-317h, and COG-LL-332h cells by 29-fold (*P* ≤ 0.001), 28-fold (*P* ≤ 0.001), and 224-fold (*P* ≤ 0.001) respectively (Figure 6A), relative to sphinganine + GT-11 with no fatty acid. C22:1-FA added to sphinganine + GT-11 increased C22:1-dihydroceramide in MOLT-4, COG-LL-317h and COG-LL-332h cells by 11-fold (*P* ≤ 0.001), 17-fold (*P* ≤ 0.001), and 35-fold (*P* ≤ 0.001), respectively, relative to sphinganine + GT-11 with no fatty acid. Presumptive intracellular elongation of C22:0-FA to form C24:0-FA, and of C22:1-FA to form C24:1-FA, was observed as indicated by concurrent elevations of C24:0- and C24:1-dihydroceramides, respectively. C18:0-FA affected dihydroceramide and ceramide levels to a much lesser extent. C22:0-FA addition consistently increased (*P* ≤ 0.001) cytotoxicity of sphinganine-alone and of sphinganine + GT-11 (Figure 6B), in all three cell lines, in a sphinganine concentration-dependent manner. As observed in CCRF-CEM cells, C18:0- and C22:1-FA minimally affected cytotoxicity. Spearman’s non-parametric rank correlation analyses of the cytotoxicity data and sphingolipid levels of each cell line revealed significant, strong positive correlations between cytotoxicity and the absolute levels of C22:0- and C24:0-dihydroceramides in MOLT-4 [(C22:0-DHCer, ρ = 0.81, *P* ≤ 0.01), (C24:0-DHCer, ρ = 0.86, *P* ≤ 0.01)], COG-LL-317h [(C22:0-DHCer, ρ = 0.79, *P* ≤ 0.02), (C24:0-DHCer, ρ = 0.88, *P* ≤ 0.004)], and COG-LL-332h [(C22:0-DHCer, ρ = 0.77, *P* ≤ 0.04), (C24:0-DHCer, ρ = 0.90, *P* ≤ 0.002)]. No other consistent strong positive or negative correlations were observed.

### C22:0-fatty acid increased 4-HPR cytotoxicity

**Figure 7:**
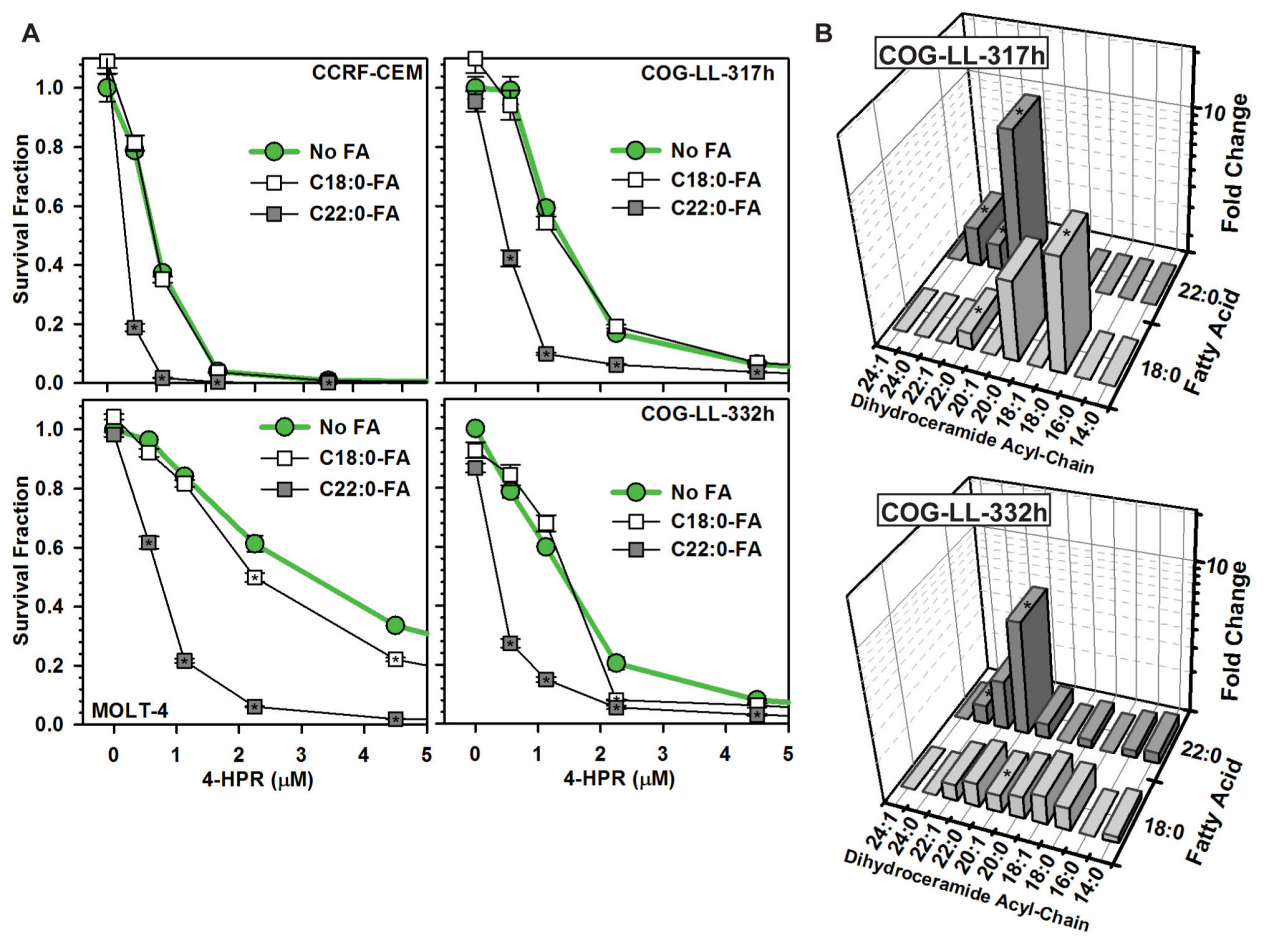
Effects of specific fatty acids on 4-HPR-induced dihydroceramide levels and cytotoxicity. *A*) Effects on dihydroceramide levels. COG-LL-317h and COG-LL-332h cells were treated with 4-HPR (1 µM) with or without C18:0- or C22:0-fatty acids (5 µM) for six hours and sphingolipids analyzed. Data were normalized to cells that received 4-HPR without fatty acid supplementation and plotted as fold change (Z-axis). Significant (*P* ≤ 0.001) differences from 4-HPR without fatty acid indicated by asterisks (*). *B*) Effects on cytotoxicity. CCRF-CEM, MOLT-4, COG-LL-317h, and COG-LL-332h cell lines were treated with 4-HPR (0-9 µM) ± C18:0- or C22:0-fatty acids (5 µM) and cytotoxicity assessed at +48 hours by DIMSCAN cytotoxicity assay. Data were normalized to controls and represented as Survival Fraction (Y-axis). Error bar, SEM. Significant (*P* ≤ 0.001) differences in cytotoxicity from 4-HPR without fatty acid are indicated by asterisks (*).

Because 4-HPR increased dihydroceramides in susceptible cell lines, but a cause and effect relationship between dihydroceramide increase and fenretinide-induced cytotoxicity remained unclear, the effects of fatty acid supplementation on 4-HPR-induced cytotoxicity and dihydroceramide levels were determined. T-cell ALL cell lines were exposed to 4-HPR ± C18:0 or C22:0-FA. Similar to the effects of specific fatty acids on sphinganine + GT-11, the addition of C22:0-FA, but not C18:0-FA, increased (*P* ≤ 0.001) 4-HPR-induced cytotoxicity in all four ALL cell lines (Figure 7A). C24:0-fatty acid increased the cytotoxicity of low 4-HPR concentrations in all cell lines (*P* ≤ 0.001); C22:1-fatty acid minimally to moderately increased 4-HPR cytotoxicity in a cell line-specific manner (Figure S4). The effects of fatty acid co-treatment on sphingolipid levels in fenretinide-treated cells were analyzed in the COG-LL-317h and COG-LL-332h cell lines. C22:0-FA addition increased C22:0-dihydroceramide levels in COG-LL-317h and COG-LL-332h cells, 10-fold (*P* ≤ 0.001) and 6-fold (*P* ≤ 0.001), respectively, over cells treated with 4-HPR-alone (Figure 7B). C18:0-FA increased (*P* ≤ 0.01) C18:0-dihydroceramide levels in COG-LL-317h cells, but not in COG-LL-332h cells (Figure 7B). However, this increase of C18:0-dihydroceramide in COG-LL-317h cells occurred in the absence of a corresponding increase in cytotoxicity (Figure 7A) suggesting that the observed effects of C22:0-dihydroceramide levels on fenretinide-induced cytotoxicity was acyl chain-specific.

## Discussion

We previously demonstrated that 4-HPR induced cytotoxicity in T-cell ALL cell lines was associated with increased levels of *de novo* synthesized long and very long acyl chain (i.e., native) dihydroceramides (2). However, a causal relationship between the increase of dihydroceramides and 4-HPR-induced cytotoxicity was not clear. The aim of the current study was to elucidate the cytotoxic potentials of native acyl chain dihydroceramides produced via *de novo* synthesis in a controlled manner and determine by correlation analysis if such cytotoxicity was due to increases in total dihydroceramide mass or to the increase of discrete dihydroceramide species. Therefore, we supplemented cells with minimally to non-cytotoxic amounts of normal sphinganine ± GT-11, a DES1 inhibitor, ± individual fatty acids (as acyl chain precursors). We then determined how the manipulation of specific dihydroceramides (via supplementation of individual fatty acids) affected fenretinide cytotoxicity. While this model lacked the specific serine palmitoyltransferase (SPT) and dihydroceramide synthase (CerS) stimulating properties of 4-HPR, it did allow for the manipulation and assessment of multiple individual dihydroceramides in isolation from other cytotoxic effects of fenretinide, such as an increase in reactive oxygen species (ROS).

Results using tracer radiolabeling and tandem mass spectroscopy demonstrated that exogenous sphinganine supplemented in non-cytotoxic amounts was incorporated into cellular sphingolipids and successfully increased levels of dihydroceramides and ceramides (Figure 2) with a sphingolipid profile that was similar but distinctive to each cell line (not shown). The addition of minimally-toxic amounts of GT-11 to sphinganine further increased most dihydroceramides at the expense of the corresponding ceramides (Figure 2 and Table 1). It was further determined using radiolabeling and tandem mass spectroscopy that, within the limitations of intracellular metabolism (i.e., shortening, elongation, and desaturation of fatty acids, and of possible cell line-specific activity of the dihydroceramide synthases), supplementation of nontoxic amounts of individual fatty acids successfully increased the level of the corresponding dihydroceramide in most cases (Figures 3 and 6, Table 1). Thus, the intracellular levels of individual native acyl chain dihydroceramides derived from *de novo* synthesis could be manipulated and their cytotoxic potentials assessed. It should be emphasized that the levels of dihydroceramides attained in this model represent levels likely not found in cancer cells in the absence of strong pharmacological stimulation of *de novo* synthesis, and not experienced in normal cells, even in dihydroceramide desaturase null mice [42]. Further, no inferences should be made on the cytotoxic potential of dihydroceramides and ceramides in other cellular pools, such as those derived from activation of various sphingomyelinases.

Correlation analysis was employed to obtain insight into the cytotoxic properties of individual dihydroceramides. Results showed that only the absolute levels of both C22:0- and C24:0-dihydroceramides strongly correlated with cytotoxicity in the CCRF-CEM, MOLT-4, COG-LL-317h, and COG-LL-332h T-cell ALL cell lines. No other consistent relationships were observed between cytotoxicity and total or other individual dihydroceramides, total or individual ceramides, or levels of the other sphingolipid species (sphinganine, sphinganine-1-P, sphingosine, sphingosine-1-P). Further, supplementation with C22:1-FA increased absolute levels of its corresponding dihydroceramide to a greater extent than did C22:0-FA without increasing cytotoxicity (Figure S5), evidencing that cytotoxicity did not correlate with total dihydroceramide mass, but rather with an increase of *specific* dihydroceramides. A limitation was that a targeted increase of dihydroceramide could not be achieved in all cases (e.g., C18:1-dihydroceramide, Figure 3) and, therefore, the cytotoxic potential of some dihydroceramides could not be assessed; further, it cannot be excluded that the cytotoxic potential of a given dihydroceramide is cancer cell line-, or cancer type-, dependent.

Results also demonstrated that the cytotoxic potentials of dihydroceramides were dependent on both acyl chain length and saturation status as, although both C22:0- and C22:1-dihydroceramides were increased to similar absolute levels, only levels of C22:0-dihydroceramide positively correlated with cytotoxicity (Figure S2). Significantly, supplementing 4-HPR-treated cells with nontoxic amounts of C22:0-FA and C24:0-FA increased 4-HPR-induced cytotoxicity (Figure 7 and Figure S4) that, in the case of C22:0-FA supplementation, was in association with increased C22:0- and C24:0-dihydroceramide levels (the direct effect of C24:0-FA supplementation on sphingolipid levels was not assayed). In contrast, C18:0-FA minimally increased 4-HPR-induced cytotoxicity despite increasing C18:0-dihydroceramide, and C22:1-FA minimally increased 4-HPR cytotoxicity despite its ability to selectively increase C22:1- and C24:1-dihydroceramides in sphinganine + GT-11 treated cells, suggesting that the increase of certain dihydroceramides, but not all dihydroceramides, may contribute to 4-HPR cytotoxicity. As the targeted increase of C22:0-dihydroceramide did not increase ROS in association with increased cytotoxicity, these results also likely exclude a role for ROS as a downstream mediator of dihydroceramide-driven cytotoxicity. These results support previous reports that 4-HPR-induced increase of ROS in susceptible cell lines occurs through a process that is mechanistically distinct from the elevation of dihydroceramides (although 4-HPR does not increase ROS in association with cytotoxicity in all cell lines) [8,43].

We had previously observed both apoptotic and non-apoptotic cell death in neuroblastoma cells treated with 4-HPR, a p53-independent agent [9]. In CCRF-CEM cells, we observed that the targeted increase of C22:0-dihydroceramide induced cytotoxicity associated with an increase in flux and levels of autophagy marker LC3B-II and caspase-dependent apoptosis (as evidenced by TUNEL-positive DNA cleavage). Interestingly, preventing DNA cleavage by pan-caspase inhibition did not significantly reduce cytotoxicity, indicating the presence of a concurrent, caspase-independent, non-apoptotic death mechanism (Figure 4). 3-methyladenine, a putative autophagy inhibitor, did not affect the cytotoxicity of C22:0-FA plus sphinganine ± GT-11 in CEM cells. Further, increased LC3B-II flux was observed in cells treated with C22:1-fatty acid, sphinganine and GT-11, suggesting that autophagy was a concurrent process that did not subserve either a strong pro-death or pro-life function in this context. The role of autophagy in dihydroceramide-associated cell death is being further investigated via the knockdown of autophagy-initiating proteins, Beclin-1 and ATG7 (work in progress).

Of note, it has recently been suggested that sphinganine level increase resulting from hydrolysis of dihydroceramides by alkaline ceramidase 2 (ACER2) is the mechanism of 4-HPR-induced cytotoxicity in tumor cells [44]. However, although the dihydroceramide profiles of cells treatment with sphinganine + GT-11 did not perfectly mimic those induced by 4-HPR in the same cell line likely due to the stimulatory effects of 4-HPR on specific CerS family members (not shown), we observed that intracellular sphinganine levels did not correlate with cytotoxicity, and that exogenous C22:0-FA increased C22:0-dihydroceramide levels and 4-HPR cytotoxicity in the *absence* of a further increase of sphinganine. Together, these findings suggest that specific dihydroceramides can mediate cytotoxicity independently of sphinganine.

It is significant that the results indicate that dihydroceramide cytotoxicity is both acyl chain length- and saturation-dependent as the individual ceramide synthase family members (CerS 1-6) each have specific fatty acyl-CoA substrate preferences. If dihydroceramides do contribute to 4-HPR cytotoxicity, we speculate that the CerS expression/activity profile of a given tumor might be a biomarker partially predictive of its response to 4-HPR. Interestingly, CerS2 was the most highly expressed (mRNA) CerS in the T-cell ALL cell lines tested (Figure S6), and the acyl-CoA preferences of CerS2 include C22:0- and C24:0-acyl chains [45]. We are currently determining how CerS2 knock-down affects the cytotoxicity of sphinganine + GT-11 ± C22:0- and C24:0-FA in these cell lines (work in progress).

In summary, the present study reports a novel method for manipulating levels of individual dihydroceramides in whole cells and evidence that dihydroceramides produced through *de novo* synthesis can confer level-dependent cytotoxicity to cancer cells in a manner dependent upon the length and saturation status of their acyl chain; specifically, C22:0- and C24:0-dihydroceramides exhibited level-dependent cytotoxicity independent of ceramide levels in four T-cell ALL cell lines. Additionally, as co-treatment with nontoxic amounts of C22:0-FA enhanced 4-HPR cytotoxicity in these cell lines in association with an increase of C22:0-dihydroceramide, the data suggest that certain dihydroceramides may contribute to the overall mechanism of 4-HPR cytotoxicity. This latter observation also suggests the possibility that addition of specific fatty acids, perhaps in a tumor or tumor type-specific manner, either through dietary or intravenous supplementation or via incorporation into drug delivery vehicles, might be employed to improve the clinical efficacy of dihydroceramide-increasing anticancer agents.

Based on these findings, we are currently evaluating the effects of an oral, organized lipid complex formulation of 4-HPR enriched with C22:0-FA acid on intra-tumor dihydroceramide levels and cytotoxicity in human pediatric T-cell ALL xenograft models [46,47].

## Supporting Information

Figure S1 **Effects of fatty acids on ceramide levels.** CCRF-CEM cells were treated with (left) sphinganine (1 µM) or (right) sphinganine (1 µM) + GT-11 (0.5 µM) and supplemented with the indicated fatty acids (5 µM) for six hours with subsequent sphingolipid analysis. To evaluate the effects resulting from addition of each fatty acid, data for (*A*) & (*B*) were normalized either 1) to cells that received sphinganine-only with no fatty acid supplementation (*A*) or, 2) to sphinganine + GT-11 without fatty acid (*B*), and plotted as fold change (Z axis) ceramide. Fatty acids are identified by x:y, where x is the number of carbons and y is the number of double bonds in the fatty acid chain (Y axis). Significant (*P* ≤ 0.05) differences are indicated by asterisks (*).
(PDF)

Figure S2 **Relationships between cytotoxicity and C22:0-, C22:1- and C24:0-dihydroceramides.** Absolute levels (X-axis) of C22:0-DHCer (*left*), C22:1-DHCer (*middle*), and C24:0-DHCer (*right*) were plotted against the Killed Fraction (Y-axis) of the respective treatment as measured using DIMSCAN. CCRF-CEM cells were treated with sphinganine (1 µM) ± GT-11 (0.5 µM) with and without fatty acid supplementation (C14:0-, C16:0-, C18:0-, C18:1-, C20:0-, C20:1-, C22:0-, C22:1-, C24:0- and C24:1-fatty acids (5 µM)). Open circles indicate treatment with sphinganine ± FA; closed circles indicate treatment with sphinganine + GT-11 ± FA. *X* and *Y* error bars are SEM. Spearman correlation coefficients (*ρ*) are shown.
(PDF)

Figure S3 **TUNEL positivity in sphinganine and/or GT-11 treated CCRF-CEM cells supplemented with C22:0-FA or C22:1-FA.** Cells were treated as indicated with sphinganine (1 µM, S), GT-11 (0.5 µM, G) and C22:0-FA or C22:1-FA. After +24 hours, cells were fixed and subsequently analyzed by TUNEL assay. C22:1-fatty acid served as a negative control for C22:0-fatty acid. Shown are singlet histograms representative of three independent experiments. Increased TUNEL positivity is observed versus similar treatment in Figure 4C. This is due to the presence of DMSO, vehicle of Boc-D-FMK, which may interfere with cyclodextrin inclusion complexes and reduce the effective fatty acid concentration.
(PDF)

Figure S4 **Effects of specific fatty acids on 4-HPR-induced cytotoxicity.** CCRF-CEM, MOLT-4, COG-LL-317h, and COG-LL-332h cell lines were treated with 4-HPR (0-9 µM) ± C18:0-, C22:0-, C22:1-, or C24:0-fatty acids (5 µM) and cytotoxicity assessed at +48 hours by DIMSCAN cytotoxicity assay. Data were normalized to controls and represented as Survival Fraction (Y-axis). Error bar, SEM. Significant (P ≤ 0.001) differences in cytotoxicity from 4-HPR without fatty acid are indicated by asterisks (*).
(PDF)

Figure S5 **Total dihydroceramide levels.** CCRF-CEM cells were treated drug vehicles (C), GT-11 (G) (0.5 µM), sphinganine (S) (1 µM), or sphinganine + GT-11. The indicated fatty acids (5 µM) were supplemented as indicated, and “No FA” indicates treatment with fatty acid vehicle. Cells were treated for six hours, followed by sphingolipid assay. Plotted are absolute total dihydroceramide levels (Z axis). Fatty acids are identified by x:y, where x is the number of carbons and y is the number of double bonds in the fatty acid chain.
(PDF)

Figure S6 **CerS mRNA levels in T-cell ALL cell lines.** Two-step RT-PCR was performed using mRNA extracted from untreated CCRF-CEM, MOLT-4, COG-LL-317h and COG-LL-332h cell lines. Data were normalized to GAPDH and calibrated to the CerS1 mRNA of CCRF-CEM cells (Y axis). Data normalized to HPRT1 instead of GAPDH were similar. Error bar, SEM. CerS3 mRNA was minimally detectable.
(PDF)
