# Supplementary figures and images for: C22:0- and C24:0-dihydroceramides Confer Mixed Cytotoxicity in T-Cell Acute Lymphoblastic Leukemia Cell Lines
Source: PLoS One. 2013 Sep 9;8(9):e74768. doi: 10.1371/journal.pone.0074768 (PMC3767634; doi:10.1371/journal.pone.0074768)

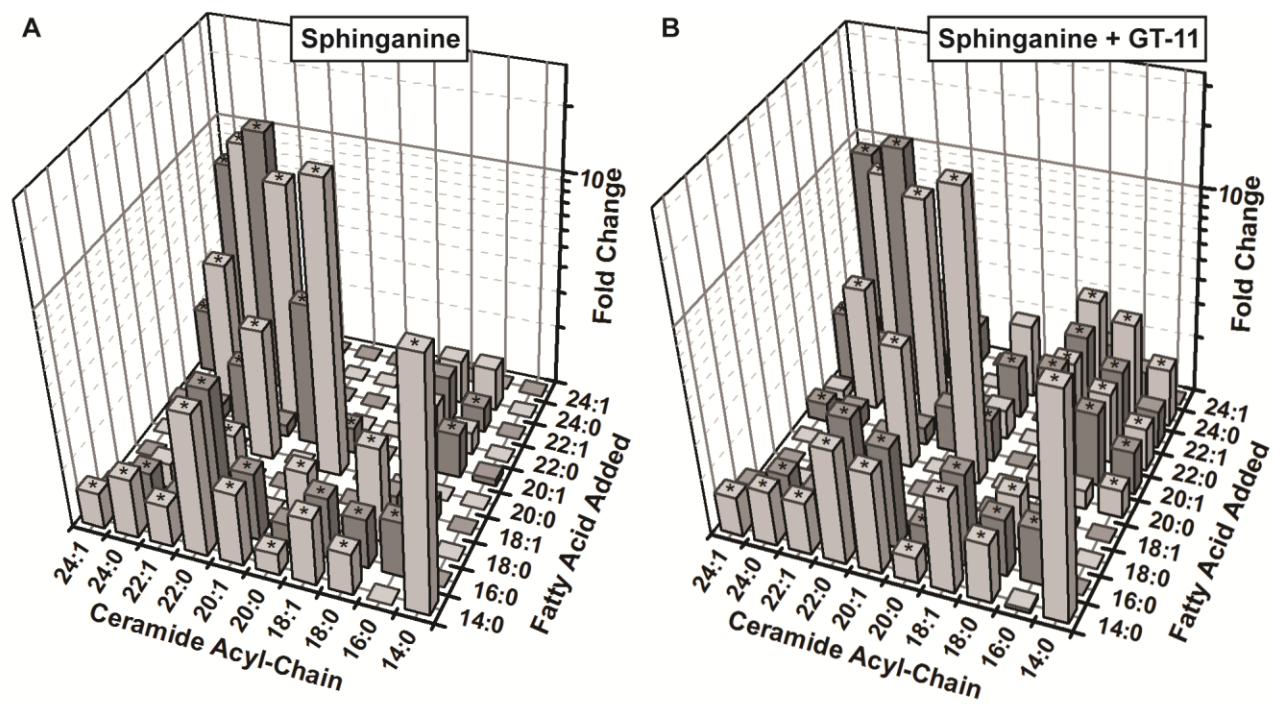

Figure S1.

Supplement: Figure S1 — Effects of fatty acids on ceramide levels. CCRF-CEM cells were treated with (left) sphinganine (1 µM) or (right) sphinganine (1 µM) + GT-11 (0.5 µM) and supplemented with the indicated fatty acids (5 µM) for six hours with subsequent sphingolipid analysis. To evaluate the effects resulting from addition of each fatty acid, data for (A) & (B) were normalized either 1) to cells that received sphinganine-only with no fatty acid supplementation (A) or, 2) to sphinganine + GT-11 without fatty acid (B), and plotted as fold change (Z axis) ceramide. Fatty acids are identified by x:y, where x is the number of carbons and y is the number of double bonds in the fatty acid chain (Y axis). Significant (P ≤ 0.05) differences are indicated by asterisks (*). (PDF) [file pone.0074768.s001.pdf]

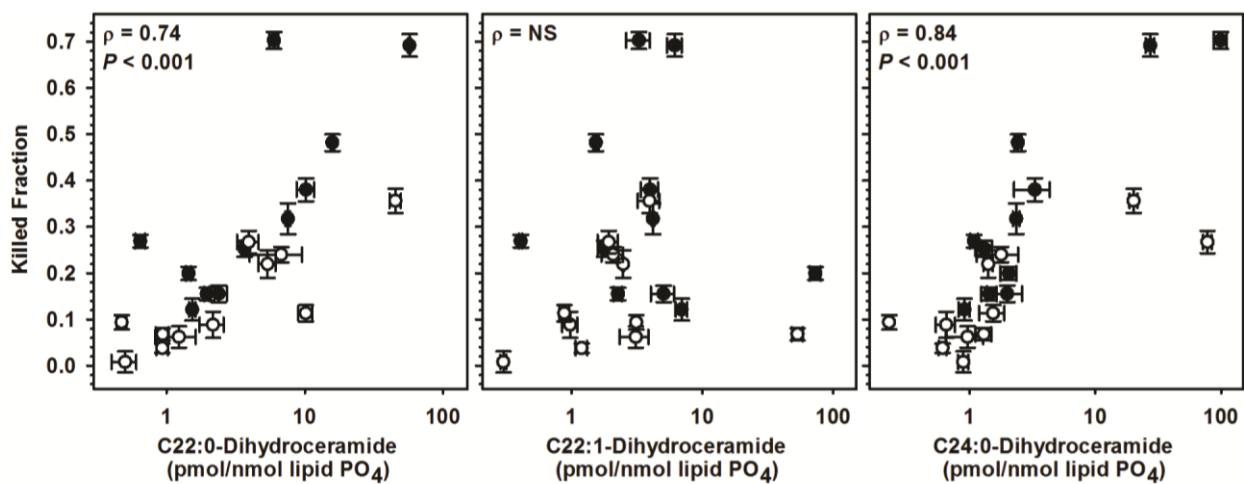

Figure S2.

Supplement: Figure S2 — Relationships between cytotoxicity and C22:0-, C22:1- and C24:0-dihydroceramides. Absolute levels (X-axis) of C22:0-DHCer (left), C22:1-DHCer (middle), and C24:0-DHCer (right) were plotted against the Killed Fraction (Y-axis) of the respective treatment as measured using DIMSCAN. CCRF-CEM cells were treated with sphinganine (1 µM) ± GT-11 (0.5 µM) with and without fatty acid supplementation (C14:0-, C16:0-, C18:0-, C18:1-, C20:0-, C20:1-, C22:0-, C22:1-, C24:0- and C24:1-fatty acids (5 µM)). Open circles indicate treatment with sphinganine ± FA; closed circles indicate treatment with sphinganine + GT-11 ± FA. X and Y error bars are SEM. Spearman correlation coefficients (ρ) are shown. (PDF) [file pone.0074768.s002.pdf]

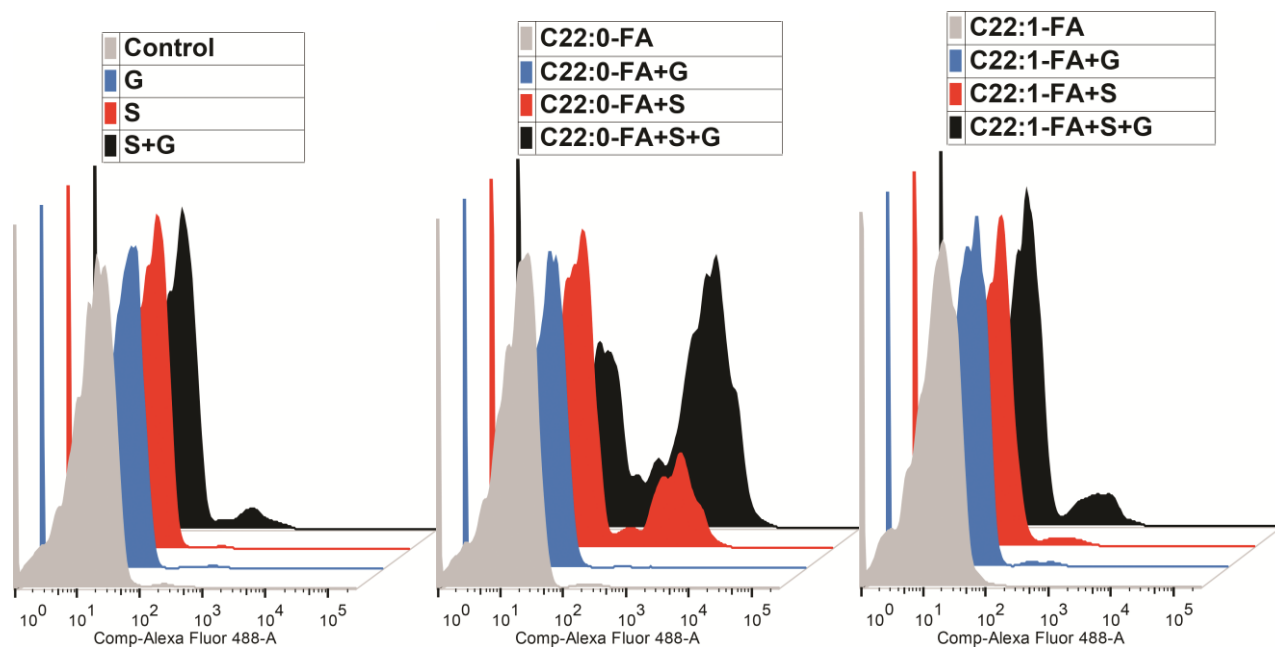

**Figure S3.**

Supplement: Figure S3 — TUNEL positivity in sphinganine and/or GT-11 treated CCRF-CEM cells supplemented with C22:0-FA or C22:1-FA. Cells were treated as indicated with sphinganine (1 µM, S), GT-11 (0.5 µM, G) and C22:0-FA or C22:1-FA. After +24 hours, cells were fixed and subsequently analyzed by TUNEL assay. C22:1-fatty acid served as a negative control for C22:0-fatty acid. Shown are singlet histograms representative of three independent experiments. Increased TUNEL positivity is observed versus similar treatment in Figure 4C. This is due to the presence of DMSO, vehicle of Boc-D-FMK, which may interfere with cyclodextrin inclusion complexes and reduce the effective fatty acid concentration. (PDF) [file pone.0074768.s003.pdf]

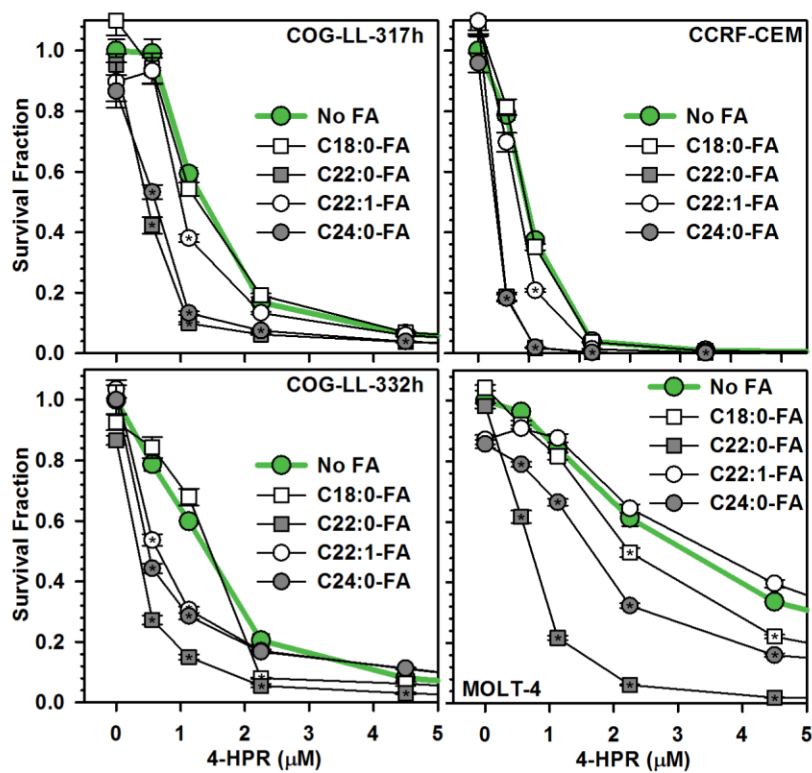

Figure S4.

Supplement: Figure S4 — Effects of specific fatty acids on 4-HPR-induced cytotoxicity. CCRF-CEM, MOLT-4, COG-LL-317h, and COG-LL-332h cell lines were treated with 4-HPR (0-9 µM) ± C18:0-, C22:0-, C22:1-, or C24:0-fatty acids (5 µM) and cytotoxicity assessed at +48 hours by DIMSCAN cytotoxicity assay. Data were normalized to controls and represented as Survival Fraction (Y-axis). Error bar, SEM. Significant (P ≤ 0.001) differences in cytotoxicity from 4-HPR without fatty acid are indicated by asterisks (*). (PDF) [file pone.0074768.s004.pdf]

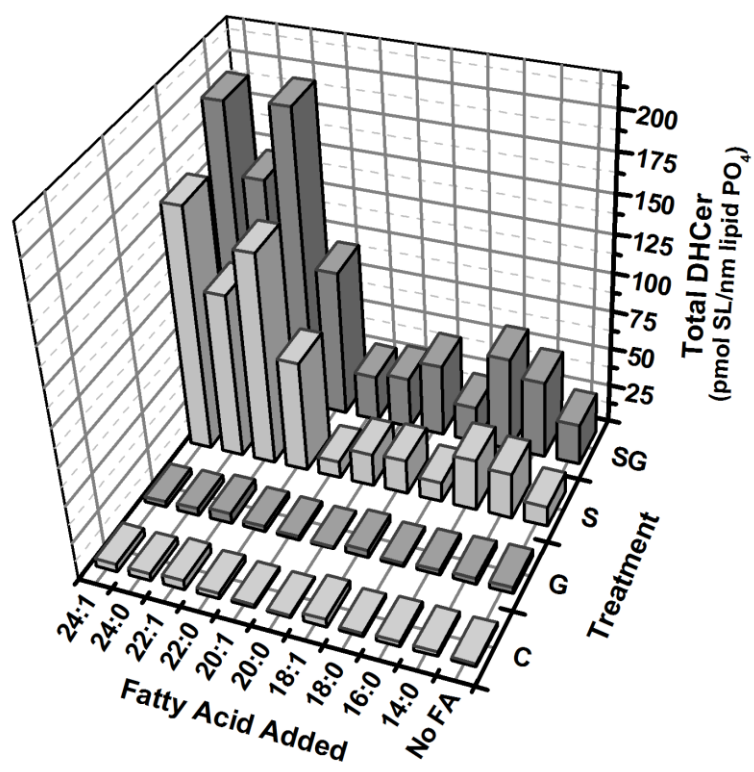

Figure S5.

Supplement: Figure S5 — Total dihydroceramide levels. CCRF-CEM cells were treated drug vehicles (C), GT-11 (G) (0.5 µM), sphinganine (S) (1 µM), or sphinganine + GT-11. The indicated fatty acids (5 µM) were supplemented as indicated, and “No FA” indicates treatment with fatty acid vehicle. Cells were treated for six hours, followed by sphingolipid assay. Plotted are absolute total dihydroceramide levels (Z axis). Fatty acids are identified by x:y, where x is the number of carbons and y is the number of double bonds in the fatty acid chain. (PDF) [file pone.0074768.s005.pdf]

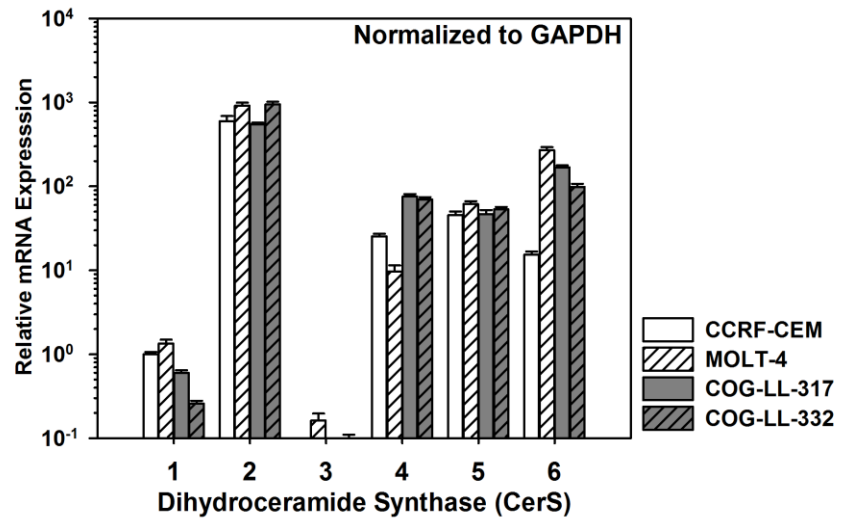

**Figure S6.**

Supplement: Figure S6 — CerS mRNA levels in T-cell ALL cell lines. Two-step RT-PCR was performed using mRNA extracted from untreated CCRF-CEM, MOLT-4, COG-LL-317h and COG-LL-332h cell lines. Data were normalized to GAPDH and calibrated to the CerS1 mRNA of CCRF-CEM cells (Y axis). Data normalized to HPRT1 instead of GAPDH were similar. Error bar, SEM. CerS3 mRNA was minimally detectable. (PDF) [file pone.0074768.s006.pdf]
